# Influence of the Conditions of Corotating Twin-Screw Extrusion for Talc-Filled Polypropylene on Selected Properties of the Extrudate

**DOI:** 10.3390/polym11091460

**Published:** 2019-09-06

**Authors:** Emil Sasimowski, Łukasz Majewski, Marta Grochowicz

**Affiliations:** 1 Department of Technology and Polymer Processing, Faculty of Mechanical Engineering, Lublin University of Technology, 20-618 Lublin, Poland; e.sasimowski@pollub.pl; 2 Department of Polymer Chemistry, Faculty of Chemistry, Maria Curie-Sklodowska University, 20-614 Lublin, Poland; mgrochowicz@umcs.pl

**Keywords:** processing, twin-screw extrusion, kneading elements, polypropylene, talc

## Abstract

The aim of the study was to determine the effect of the application of processing screws with a modified test segment in a corotating twin-screw extruder on selected properties of talc-filled polypropylene extrudate. The test segment was built of trilobe kneading elements and its design modifications refered to changing the distance between the kneading elements and the angle of positions of kneading elements that are relative to each other. The performed tests included the production of extrudate with various degrees of talc-filling using five design solutions of the test segment and then measurements of selected properties, such as tensile strength, elongation at maximum tensile stress, and melt flow rate. Structural studies using scanning electron microscope (SEM) and differential scanning calorimetry (DSC) were also carried out. The study includes not only the description of experimental results but also the determination of empirical models describing the dependence of the properties of the obtained extrudate on the conditions of the extrusion process and the design features of the test segment.

## 1. Introduction

Polypropylene is one of the most commonly used polymer materials, thanks to its properties such as good stiffness, high melting point, chemical resistance, easy processability and very good mechanical strength to density ratio [1,2,3,4]. These properties make it an attractive material for many engineering applications. Currently, due to increasingly higher requirements in terms of both the properties of the materials used and the economy of production, the use of unmodified materials has become insufficient or unprofitable [4,5,6,7,8,9,10]. A very strong commercial interest in polypropylene initiated intensive research of its composites. Initially, additives introduced to polypropylene were intended primarily to reduce the cost of production and improve the degree of crystallinity and dimensional stability of products. Polypropylene is characterized by a significant processing shrinkage rate, even up to 3%. The available literature includes numerous papers on composites based on polypropylene matrix, reinforced with solid particles, fibres, organic materials and layered inorganic fillers. Their introduction is aimed at improving the functional properties and obtaining the desired stiffness, thermal resistance, mechanical properties, corrosion resistance or gas permeability and reducing the manufacturing costs [4,7,11]. 

The most commonly used fillers in polymer plastics processing are powdered minerals from the group of phyllosilicates. Fillers introduced into polypropylene are carbonates, wollastonite, kaolin, montmorillonite, zeolite, mica and talc. Talc is the most popular polypropylene (PP) filler due to its low price, common occurrence and favourable lamellar structure [1,3,6,7]. Talc is a magnesium hydroxysilicate(Mg_3_Si_4_O_10_(OH)_2_), which is a result of hydrothermal degradation of magnesium-rich metamorphic rocks. The crystalline structure has a layered composition, in which magnesium oxide octahedra are located between silica tetrahedra [12,13,14,15]. Such a structure provides a neutrally charged crystal system, where all vacancies remain filled and no surface charges are observed. As a result, the only bond between the individual layers of talc is the Van der Waals force, which defines talc as the softest of minerals, which opens up Mohs’ hardness scale. A neutral electric charge, apart from its negligible hardness, induces specific chemical properties of the surface. The top layer of talc, as one of the few clay minerals, has hydrophobic properties that lead to poor wettability and significantly reduce dispersion in water media. The chemical properties described above, combined with a significant specific surface area of talc powder particles, allow to obtain a strengthening effect when used as a filler for thermoplastic polymers [14,16,17]. It is worth noting that despite good compatibility between talc and polypropylene, many authors of research papers decide to use a compatibilizer to ensure stronger interactions at the interface [8,18,19]. 

Initially, talc was used only as a dimensional stabilizer for polypropylene products or as a filler to reduce production costs. The research has shown that in appropriate conditions it is possible to obtain the effect of strengthening PP with talc, which improves the mechanical properties of the obtained composites. In order to improve the composite properties, the following factors should be taken into account: polymer structure, amount and type of filler, strength of adhesive interactions at the matrix/filler interface, but first of all the quality of filler distribution in the polymer matrix. It depends on the technological parameters of the processing and design features of the plasticizing system of the processing machine [7,20,21]. The issue of proper mixing and distribution of particulate matter in the polymeric matrix, despite a number of studies carried out in this field, still remains valid. This state of affairs is partly due to the complicated procedure of assessing the quality of mixing, which is usually based on the observation of local cross-sections and the adaptation of the obtained results to the whole volume of the tested elements. The strong tendency of mineral fillers, including the most frequently used talc, to form agglomerates displaying definitely unfavourable influence on the properties of the composite [1,5,22] should also be emphasized. Therefore, for the production of composites based on polymer matrix, corotating twin-screw extruders, characterized by very good mixing efficiency in comparison to single screw extruders, are most often used. This is dictated by the specificity of the melt mixing process, where initially agglomerates of the mineral filler are formed in the melt section of the plasticizing system; the agglomerates then have to be crushed and mixed with the polymer matrix [23,24]. Mixing in the extruder′s plasticizing system is divided into shear mixing (also called shredding or dispersion mixing) and distribution of the mixture components. Processes with positive impact on the homogenization degree of the processed composition, such as shear intensity controlled by the configuration of the screw kneading segments or screw rotational speed, may negatively influence the properties of the matrix itself, causing degradation of polymer chains and deterioration of composition strength, especially during multiple processing [25,26,27]. Therefore, the selected configuration of the screw elements should provide a suitable residence time range for the material to be processed in the plasticizing system, a shear rate range and a pressure variation course. This is particularly important because the above-mentioned factors directly translate into the quality of homogenization of the polymer and mineral filler, which is extremely important for the extrusion of polymer nanocomposites [28,29].

The segment of the screw corresponding to intensive mixing and plasticizing is usually composed of kneading elements in the form of one-, two- or three-lobe cams. Two-lobe cams are most commonly used. The intensive mixing segment composed of kneading elements is characterized by three basic geometric features: the length of the segment, the angle of the position of kneading elements in relation to each other and the width of a single cam element. In addition, if the angle between the cooperating kneading elements is different from zero, the direction of the apparent screw flight defined by the cam lobes can be determined [25,30]. On the basis of the literature review, it can be concluded that the influence of the setting of two-lobe elements on the course of the extrusion process has been well known and was the subject of intensive research as early as in the 1990s [31,32,33]. However, in 2015, a doctoral dissertation [34] was published on the influence of the application of a new design solution for the segment of intensive plasticizing and mixing of materials using two-lobe kneading elements on the characteristics of the extrusion process and properties of the composites obtained. According to the patent description [35], the new solution consists in introducing passive spacer elements in the shape of sleeves between the kneading elements. By replacing spacer elements, it is possible to change the distance between the kneading elements—an additional parameter describing the segment.

The available literature, however, does not deal with the configuration of intensive mixing segments composed of trilobe elements, let alone with the parameter of distance between them, and their influence on the characteristics of extrusion process and properties of the composites obtained. Therefore, the aim of this paper was to investigate the influence of the design solution of the segment of intensive mixing and plasticizing, consisting of trilobe kneading elements, on selected mechanical and structural properties of the obtained polypropylene/talc extrudate.

## 2. Experimental

### 2.1. Test Stand

**Figure 1:**
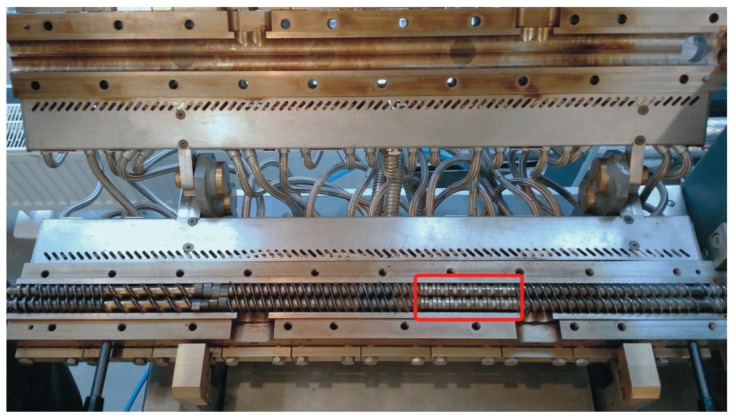
View of an open cylinder of the plasticizing system of an extruder with screws in one of the tested configurations, with a border-marked test segment.

**Figure 2:**
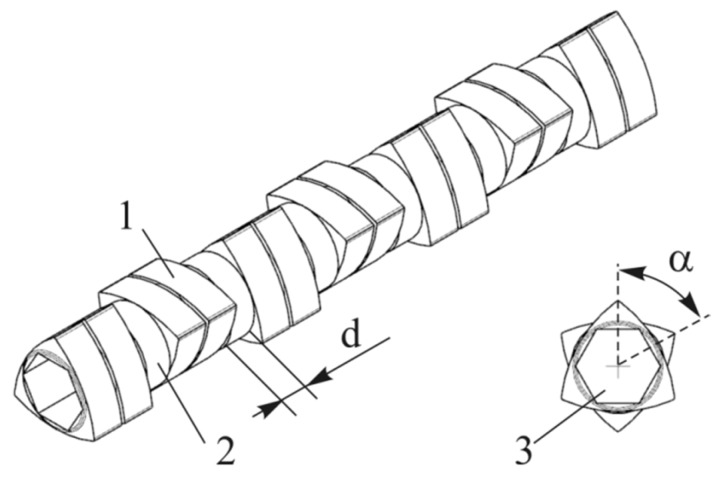
Scheme of the test segment 1—triple-lobe kneading element; 2—spacer; 3—hexagon drill for the screw core; d—distance between kneading elements; α—angle between the kneading elements.

**Figure 3:**
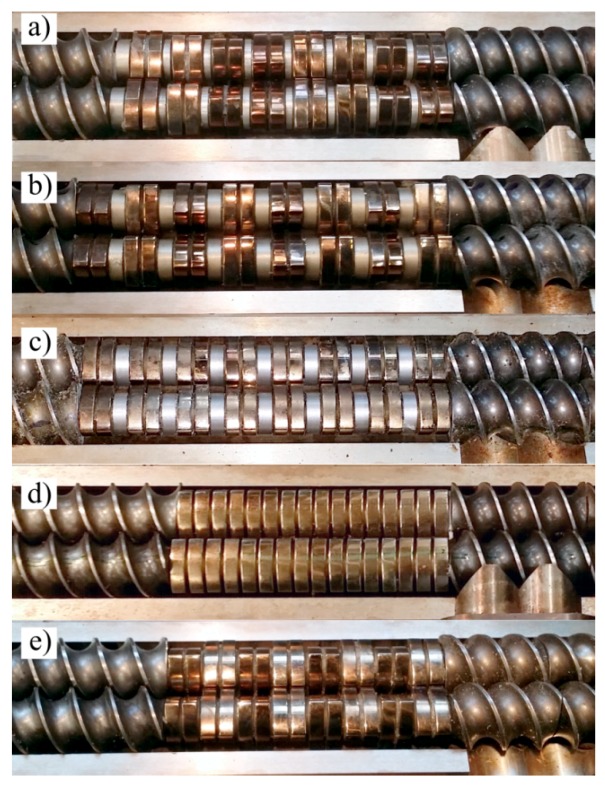
View of the design solutions of the test segment: (**a**) d = 2.5 mm α = 30°; (**b**) d = 4.5 mm α = 60°; (**c**) d = 4.5 mm α = 0°; (**d**) d = 0.5 mm α = 0°; **(e**) d = 0.5 mm α = 60°.

The extrusion process was carried out with the use of an EHP-2x20 Sline corotating twin-screw extruder by Zamak Mercator (Skawina, Poland). It was equipped with a plasticizing system with a horizontally split cylinder allowing access to segmented processing screws with a diameter of D = 20 mm and working part to diameter ratio of L/D = 40. The location of the test segment on the processing screws is shown in Figure 1. The applied test segment creating the zone of intensive plasticizing and mixing of the polymers was made of trilobe kneading elements—cam discs joined in pairs with the total width of 10 mm. The front profiles of five design solutions of the tested segment used in the test were presented in Figure 2. Presented design solutions differ in the angle between the interacting kneading elements αand the distance d between the individual elements. The direction of inclination of the apparent screw flight of the successive ridges of kneading elements was clockwise or neutral (α = 0°). Individual design solutions of the test segment subjected to the tests are shown in Figure 3. The length of the segment, depending on the applied solution, changed from 4D to 5.5D, while its location—distance from the extruder filling opening—was constant and amounted to 9.5D. 

A 22.8 mm × 1.4 mm rectangular section nozzle for manufacturing polymer strips was used for the extrusion. The extrudate was cooled in a cooling tank.

### 2.2. Research Programme and Methodology

The research was carried out in accordance with the accepted multi-factor two-level complete design, which was supplemented with additional experiments in the centre of the plan. The following independent variables have been adopted—adjustable process conditions: angle between the cooperating kneading elements α = 0–60° (direction of inclination of the apparent screw flight was clockwise), distance between kneading elements *d =* 0.5–4.5 mm, screw rotational speed *n* = 100–400 min^−1^, mass fraction of input talc in polypropylene *u* = 10–16%. The experimental designs are presented in Table 1. The measurements were carried out in five repetitions.

In the experiments, Moplen EP440G polypropylene was used and produced in granular form by Lyondellbasell S.A. [34] It is a heterophasicimpact copolymer of PP with PE. Finntalc M15 talc (hydrated magnesium silicate) by Mondo Minerals (Amsterdam, Netherlands) [35] was applied as filler. According to the manufacturer′s data, the talc powder particle size does not exceed 17 μm and the content of particles smaller than 2 μm is 18%. Average particle size is 4.5 μm.

The temperature in all heating zones of the plasticizing system and extrusion head was 195 °C. It was selected on the basis of literature on extrusion of polypropylene in question [34,35,36,37,38,39,40,41]. The extruder was gravity fed, a constant level of PP/talc mixtures was maintained in the charging hole of the extruder during the extrusion.

The experimental tests carried out on the properties of the obtained extrudate included the determination of:

Melt flow rate (MFR) (190 °C/2.16 kg) (g/10min). The measurement was performed with an extrusion plastometer CEAST model MeltFlow TQ6841 in accordance with EN ISO 1133-1 method A, tensile strength σ (MPa), relative nominal elongation at maximum tensile stress ε (%), using a Zwick/RoellZ010 test machine (Kennesaw, GA, USA) according to EN ISO 527-2/1A/50 at a tensile speed of 50 mm/min.

Listed *MFR*, σ andεvalues were the observed dependent variables adopted in the studies. 

The applied research methodology allowed for the determination of mathematical models describing the variability of the propeies of extrusion as a function of independent variables (present conditions of the process). The analytical form of the sought-after empirical models corresponded to the polynomials of many variables, consisting of the following segments: constant value, linear segments, binary and ternary interaction segments (Equation (1)). Where *Y* is the predicted response value (Y stands for *MFR*, σ,ε*),* a_0_ is a constant value and *a*_x_ is a regression factor. When the models revealed excessive nonlinear effect, an additional term accounting for a pure curvature was included.

(1) $$Y\left(\alpha ,\;d,\;n,\;u\right)=a_{0}+a_{1}\alpha +a_{2}d+a_{3}n+a_{4}u+a_{12}\alpha d+a_{13}\alpha n+a_{14}\alpha u+a_{23}\mathrm{dn}+a_{24}\mathrm{du}+a_{34}\mathrm{nu}\;+a_{123}\alpha \mathrm{dn}+a_{124}\alpha \mathrm{du}+a_{134}\alpha \mathrm{nu}+a_{234}\mathrm{dnu}.$$

DSC tests of the obtained extrudate were also performed using a DSC 204 F1 Phoenix differential scanning calorimeter (NETZSCH, Günzbung, Germany) and NETZSCH Proteus data software for processing. The crystallinity degree *Xc* and melting enthalpy Δ*H* were determined, along with melting point *T*_m_ and crystallisation temperature *T*_c_ of the obtained extrudate. The crystallinity degree was calculated from the relation *X*_c_ = (ΔH/(1 − *u*) × Δ*H*_100%_) × 100%) assuming that for PP Δ*H*_100%_ = 209 J/g. DSC curves were recorded in the system of heating (I) from −100 to 240 °C (at 10 K/min), cooling from 240 to −100 °C (at 10 K/min), heating (II) from −100 to 240 °C (at 10 K/min). The analyses were carried out in an inert gas (argon) atmosphere at a flow rate of 20 mL/min. The samples were analysed in aluminium crucibles with a pierced lid.

A Nova NanoSEM 450 scanning electron microscope (Hillsboro, OR, USA) by FEI was used to observe the structure of extrudate samples. It is a high-resolution SEM microscope working in high and low vacuum, designed to study the structure and also phase and chemical composition in a wide range of magnifications. Extrudate samples were prepared for microscopic observation by making their fractures perpendicular to the direction of the extrusion. In order to achieve a brittle breakthrough, the strips were cooled in liquid nitrogen for one minute before breaking. The observation was performed with the use of back-scattered electron detector to work in low vacuum (GAD), which reveals areas differing in chemical composition, and the secondary electron detector to work in low vacuum working on the principle of direct electron detection (LVD), which reveals the topography of the surface.

DSC measurements and microscopic observations of the structure were carried out for selected samples of the obtained extrudate. The pairs of samples differing in the value of one of the independent variables were compared, while maintaining other process conditions. For comparison purposes, the initial granules of polypropylene without filler were also examined.

## 3. Test Results and Discussion

The results of investigations into the properties of talc-filled polypropylene extrudate obtained by twin-screw extrusion are presented in Table 1. It contains average values of the studied dependent variables (MFR, σ, ε) as determined in the individual experimental design layouts and their standard deviations. On the basis of the collected measurement results, regression coefficients were estimated for empirical models expressing causal relationships between observed dependent variables and a set of independent variables—adjustable process conditions. The results of experimental tests were statistically elaborated using analysis of variance. The verification of the correctness of the construction of empirical models and statistical evaluation of individual segments of the determined regression equations were carried out. Pareto charts of standardized effects were used to statistically assess the influence of particular independent variables on the properties of the obtained extrudate. It allows to present in an illustrative way the influence of particular segments of regression equations on the value of the modelled dependent variable—the properties of the extrudate. The absolute values of standardized effects presented on the diagrams, exceeding the vertical line corresponding to the assumed level of significance p = 0.05 are considered statistically significant.

### 3.1. Melt Flow Rate

**Figure 4:**
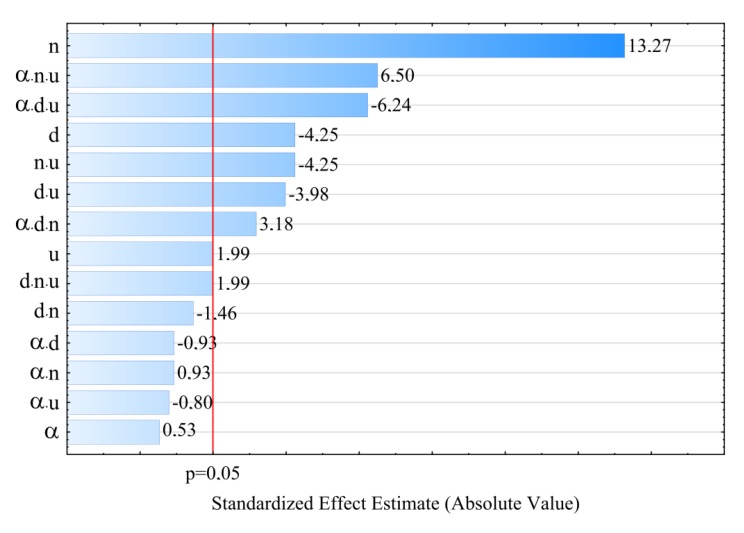
Pareto plots of the standardized effects of empirical model MFR, the vertical line in the plot corresponds to the arbitrarily chosen level of significance (p = 0.05).

**Figure 5:**
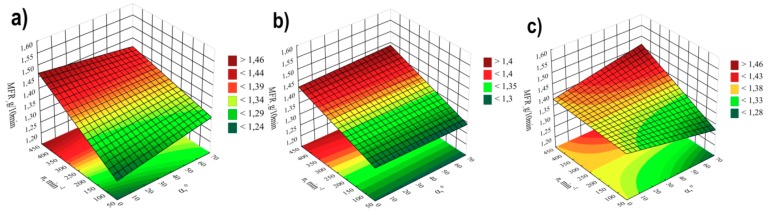
MFR response surface plots based on screw rotational speed *n* and angle of rotation of the kneading elements αwith a talc content of (**a**) *u* = 10%; (**b**) *u* = 13%; (**c**) *u* = 16%.

**Figure 6:**
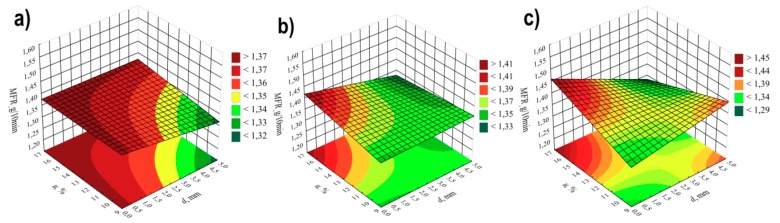
MFR response surface plots based on talc content *u* and distances between the kneading elements *d*, at the value of the angle between the kneading elements α of: (**a**) α = 0°; (**b**) α = 30°; (**c**) α= 60°.

The results of melt flow rate modelling are shown in Figure 4, Figure 5 and Figure 6. The empirical model in the form of a polynomial of four variables α, *d, n, u,* was adopted, in which, apart from linear and binary interaction segments, also ternary interaction segments are included. The results of statistical analysis of the adopted model are presented in Table 2. Melt flow rate (MFR) has shown that the most significant impact on its value is exerted by the screw speed *n* and the distance between the kneading elements *d.* The highest *MFR* increase of 0.26 g/10min (21%) was obtained during the tests as a result of the increase of screw speed from 100 to 400 min^−1^ in comparable (with the same values of other independent variables) experimental design layouts 1 and 3.A significant influence of ternary interactions is also noticeable, especially in the following areas: α*nu* (positive effect), α*du* (negative effect), and α*dn* (positive effect). The interactions *nu, du* (negative effects) are also statistically significant. Increasing the screw rotational speed and thus the shear rate of the processed material causes a gain in the MFR value. This is a consequence of increasing shear stress, which causes changes in the structure of the processed material [42,43,44]. This is particularly important in the case of polypropylenes with low *MFR* values below 3g/10min, where polymer degradation occurs due to higher thermal and mechanical loads in the intensive plasticizing and mixing zone [45]. 

Increasing the distance between the kneading elements *d* decreases the shear stress to which the processed material is subjected and consequently reduces the increase in *MFR*. Lower values of this indicator were also observed when the talc content increased along with the gain in rotational speed *n* or distance *d*. Concurrently, increasing the value of the angle αwhen the speed *n* and the distance *d* or the talc content *u* are increased at the same time, causes the gain in the *MFR* value.

A similar effect of talc content on the value of MFR was found in studies [38,39].

### 3.2. Tensile Strength

**Figure 7:**
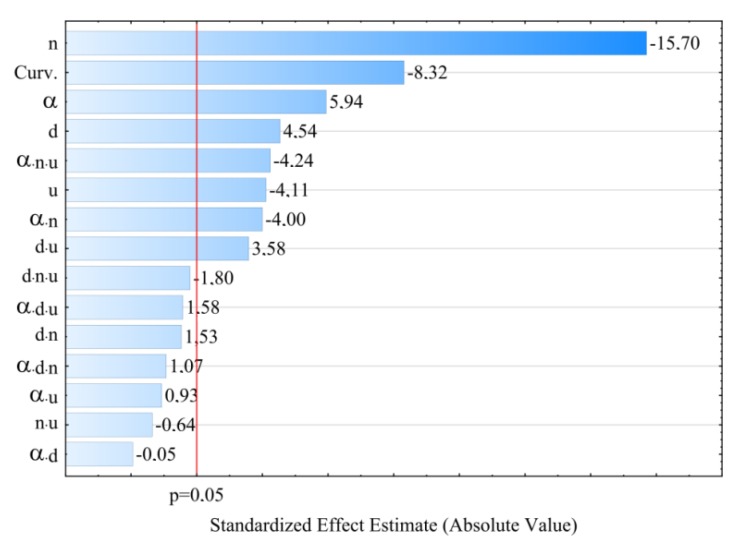
Pareto plots of the standardized effects of empirical model of tensile strength *σ*; the vertical line in plot corresponds to the arbitrarily chosen level of significance (p = 0.05).

**Figure 8:**
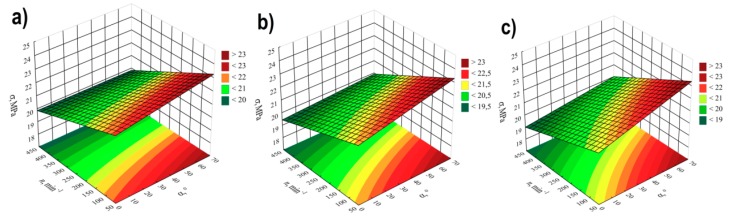
Response surface plots for tensile strength *σ*based on screw rotational speed *n* and angle between the kneading elements α, with a talc content of (**a**) *u* = 10%; (**b**) *u* = 13%; (**c**) *u* = 16%.

**Figure 9:**
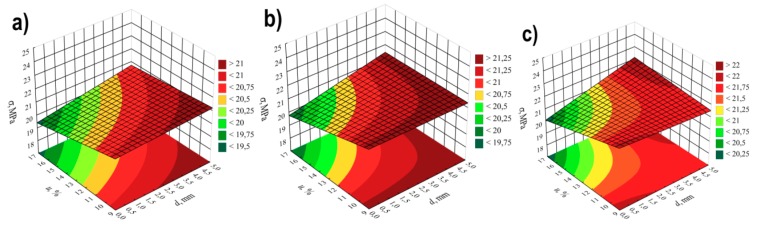
Response surface plots for tensile strength *σ*based on talc content *u* and distance between the kneading elements *d*, at the value of the angle between the kneading elements α equal to: (**a**) α = 0°; (**b**)α = 30°; (**c**) α = 60°.

On the basis of the conducted research, it was stated that the greatest impact on the tensile strength σof the obtained extrudate is exerted by the screw rotational speed *n* and angle between the kneading elements α (Table 3 and Figure 7). For modelling, polynomials were used, taking into account binary and ternary interactions, apart from linear segments. The negative effect of increasing the speed of screws on their tensile strength is associated with the partial degradation of the material in the extrusion process. At high speeds of processing screws, the processed material is sheared at high rates, which results in its degradation*—*reduction of molecular weight, the outcome of which, among others, is observed increase of *MFR* value and deterioration of strength properties. The highest observed decrease in tensile strength caused by the increase in screw rotational speed from 100 to 400 min^−1^ was 3.3 MPa (14%) in comparable experimental design layouts 14 and 16. A significant reduction in strength by 2.7 MPa (12%) also occurred between the experimental design layouts 1 and 3, 9 and 11, and 10 and 12. Increasing the angle between kneading elements α and the distance between kneading elements *d* results in an increase in tensile strength as a consequence of reduced shear of the processed material. Together with the increase in the angle α value from 0° to 60°, the maximum gain in tensile strength by 2.2 MPa (11%) and 2.1 MPa (10%) between the comparable experimental design layouts 2 and 10 as well as 6 and 14, respectively, was obtained in the tests. These differences occurred at the highest tested content of talc. Increasing the content of talc *u,* causes a deterioration of tensile strength (negative effect) by up to 1.9 MPa (9%) (experimental design layouts 1 and 2). With the gain in its content, it has a positive impact on the strength together with increasing the distance *d* between kneading elements, which is confirmed by the positive interaction between these factors. The modelling results also showed the occurrence of statistically significant interactions between α*n* and *u*, and between α and *n.* The response plots showing the relationship between the variables listed are shown in Figure 8 and Figure 9.

The statistical analysis showed that the model of tensile strength has a negative effect of the curvature (Figure 7, Table 3). Because of the experimental design type chosen, only the detection of such an effect was possible. The exact estimation of coefficients of the pure quadratic terms could not be performed.

### 3.3. Elongation at Tensile Strength

**Figure 10:**
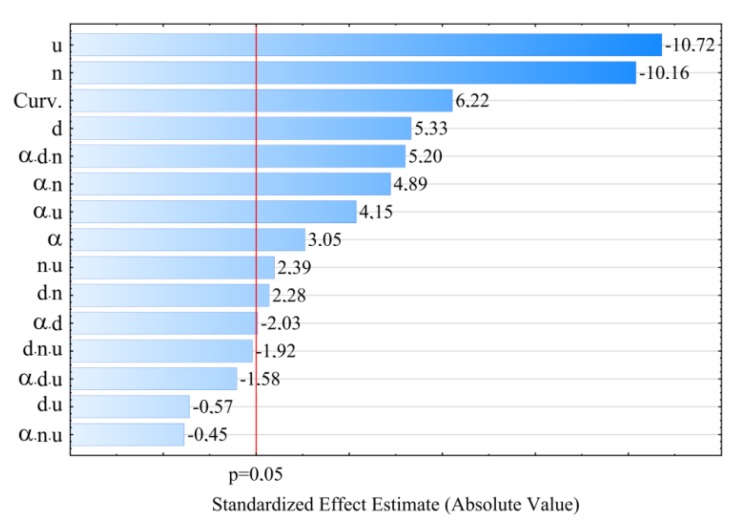
Pareto plots of the standardized effects of empirical model of elongation at tensile strength ε, the vertical line in plot corresponds to the arbitrarily chosen level of significance (p = 0.05).

**Figure 11:**
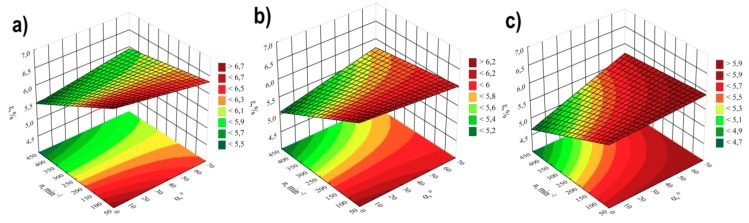
Response surface plots of elongation at tensile strength *ε*based on screw rotational speed *n* and angle of rotation of the kneading elements *α*, with a talc content of (**a**) *u* = 10%; (**b**) *u* = 13%; (**c**) *u* = 16%.

**Figure 12:**
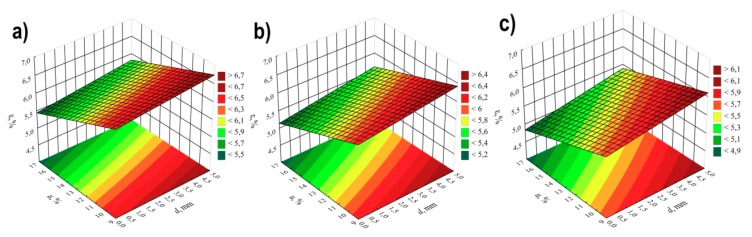
Response surface plots of elongation at tensile strength *ε*based on talc content and the distance between the kneading elements *d* at a screw speed *n* equal to: (**a**) *n* = 100 min^−1^; (**b**) *n* = 250 min^−1^; (**c**) *n* = 400 min^−1^.

For modelling the variability of the elongation of extrudate samples at tensile strength ε, the polynomial of four variables was used, namely α, *d, n, u* with linear elements, elements of two-factor interactions and elements of three-factor interactions. The results of statistical analysis of the influence of extrusion conditions on the elongation of extrudate samples are presented in Table 4 and Figure 10. The obtained response surfaces presenting the relationships between the variables listed above are presented in Figure 11 and Figure 12. It was found that the highest influence on the elongation of the samples was exerted by the content of talc *u* and the rotational speed of the screws *n.* Similarly to tensile strength, the negative effect of increasing screw speed on elongation may be interpreted as a consequence of partial degradation of the material due to increased shear rate. The highest observed decrease in elongation at tensile strength caused by the increase in screw speed from 100 to 400 min^−1^ was 1.1% in comparable experimental design layouts 6 and 8, 1 and 3, as well as 5 and 7, which corresponds to changes by 19%, 17% and 15%, respectively. On the other hand, increasing the content of talc *u* in the tested range reduces the elongation of the extrudate by a maximum of 1.4% (21%) between comparable experimental design layouts 1 and 2. The influence of distance between kneading elements *d* and angle between the kneading elements α is positive. Increasing distance *d* and angle α reduces the shear intensity of the processed material. The maximum increase in elongation due to the gain in distance *d* from 0.5 to 4.5 mm was 0.9% (18%) in comparable experimental design layouts 2 and 6. The same maximum increase in elongation of 0.9% (18%) was observed as a result of an increase in angle α from 0° to 60° between comparable experimental design layouts 8 and 16. The modelling results also showed the presence of a statistically significant interaction between α, *d* and *n*, as well as α and *n*, and also α and *u.* Also, statistically significant are the interactions between factors *n* and *u*, *d* and *n*, as well as α and *d*, although their impact is significantly smaller. Statistical analysis also showed the occurrence of curvature of model functions (Figure 10, Table 4).

### 3.4. Structure of the Extrudate

**Figure 13:**
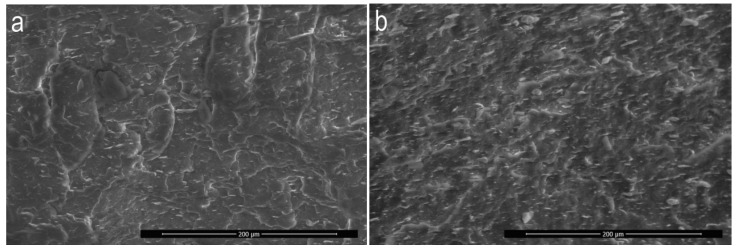

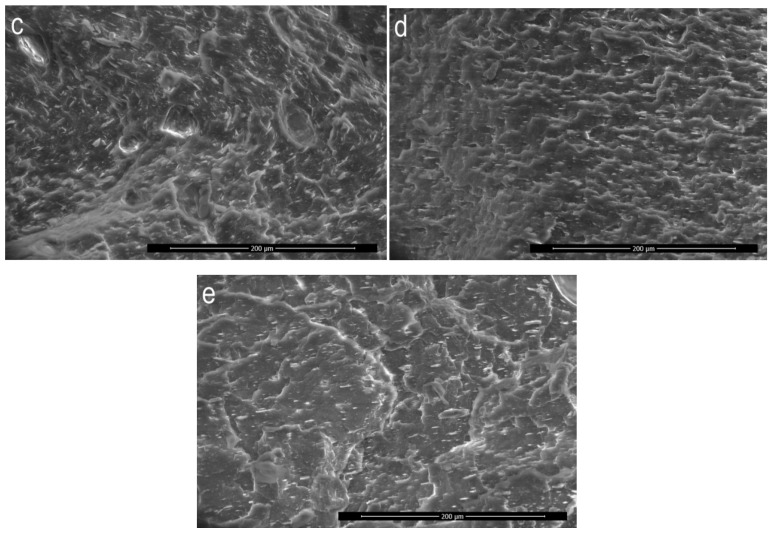
SEM images of microstructure for selected experimental design layouts: layout 13-(**a**); layout 14-(**b**); layout 12-(**c**); layout 10-(**d**); layout 6-(**e**).

For microscopic observations, samples of extrudates produced according to five experimental design layouts were selected. The samples were compared in pairs and were supposed to allow to evaluate the influence of particular variable factors (*n, d,* α*, u*) on the obtained microstructure image. Many microscopic images were used for detailed analysis of the structures, but in Figure 13a–e only micrographs of selected samples were presented, which were considered to be representative measurement samples and characteristic for the given experimental design layout. 

**Figure 14:**
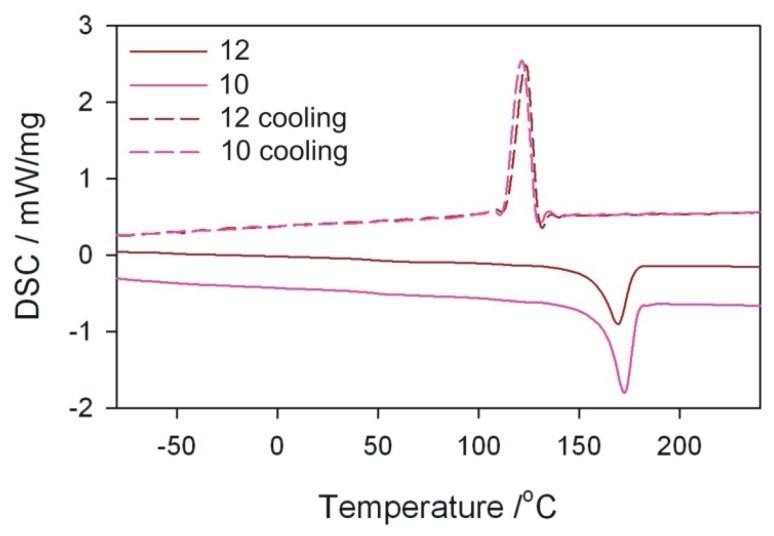
DSC curves for extrudate samples–layouts 10 and 12. Sample designations as in Table 1.

All samples observed under the microscope showed uniform distribution of talc particles in the polypropylene matrix. No unfavourable agglomerates of talc plates were found and none of the particles visible in the pictures exceed the upper dimensional limit of 17 µm given by the manufacturer. The samples of the extrudate obtained according to the experimental design layouts 13 (Figure 13a) and 14 (Figure 13b) differed in the content of talc *u* and were obtained under the same other conditions of extruding. Differences in the appearance of the microstructure are difficult to observe in microscopic images except for a significantly higher concentration of the filler for layout 14. In both cases, no characteristic orientation of talc plates was found, which can be arranged both perpendicularly and parallel to the direction of extrusion. Linear dimensions of the talc plates do not exceed 15µm. The influence of screw speed *n* on the microstructure image of PP/talc composites can be evaluated by comparing the images obtained for layouts 12 (Figure 13c) and 10 (Figure 13d). As before, no preferred orientation of talc plates was observed. On the other hand, a seemingly higher concentration of talc for layout 12 can be observed at a screw speed of 400 rpm. This is the result of the intensive grinding of the filler particles, which results in more cross-sections of talc particles in the photographs, but with significantly smaller dimensions, up to a maximum of 10 µm. For comparison, the dimensions of filler particles for extrudate 10 are up to 14 µm. Analysing the influence of the angle between the kneading elements α based on SEM images of layouts 14 and 6*,* preferred orientation of the plates was observed for layout 6 (Figure 13e) for which the angle α was 0°. The vast majority of the plates are oriented parallel to the extrusion direction, showing only the lateral edge, while the plate surfaces were observed rather occasionally. Linear dimensions of the cross-sections of the filler reached 17 µm, which may indicate limited possibilities of dispersion mixing of this configuration of the test segment. The effect of changing the distance between kneading elements *d* on the microscopic structure can be observed on the example of the extrudate obtained according to layouts 14 and 10. The sample of extrudate of layout 10 is characterised by a much better fragmentation of talc particles. Although the maximum linear dimensions of the particle cross-sections in the two samples are similar, the structure obtained for layout 10 remains clearly more fine-grained. It is likely that the introduction of distance *d* resulted in the creation of areas with reduced shear intensity between the kneading elements, thus reducing the grinding efficiency of the processed polymer composition and the grinding efficiency of the suspended solid particles.

### 3.5. DSC Analysis

**Figure 15:**
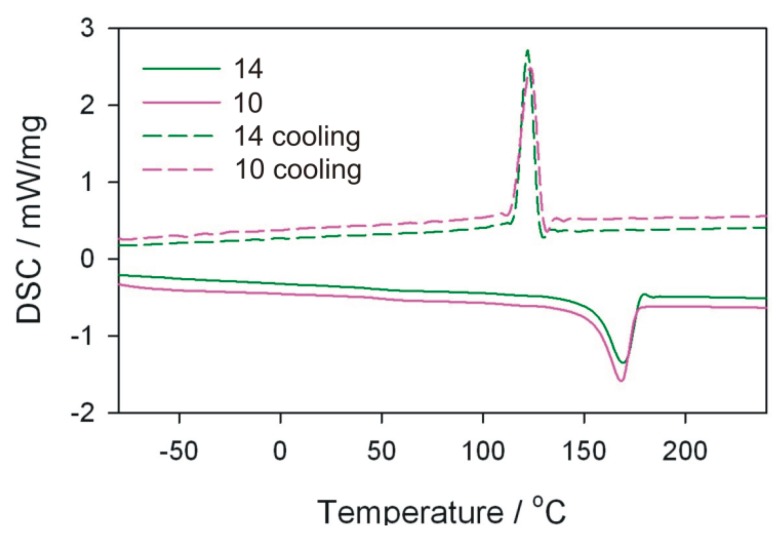
DSC curves for extrudate samples–layouts 10 and 14. Sample designations as in Table 1.

**Figure 16:**
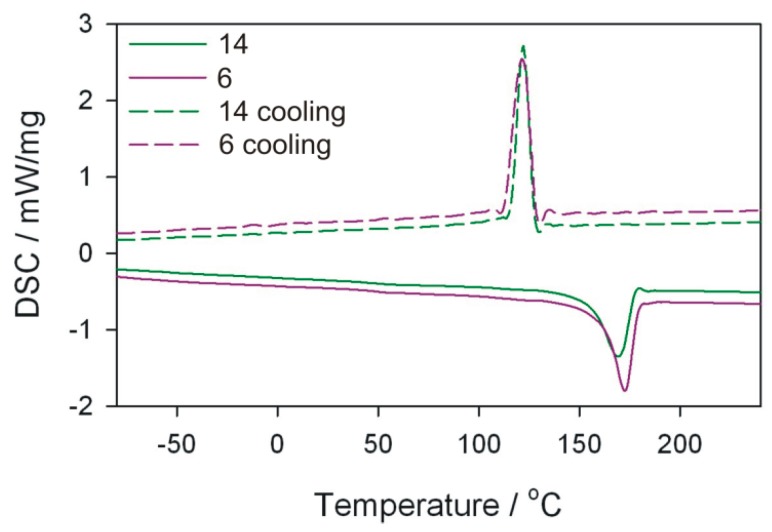
DSC curves for extrudate samples–layouts 6 and 14. Sample designations as in Table 1.

**Figure 17:**
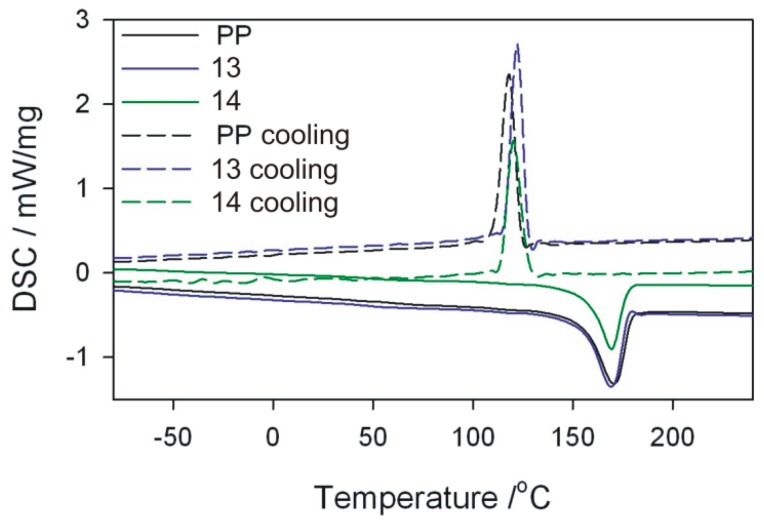
DSC curves for PP sample and extrudate samples–layouts 13 and 14. Sample designations as in Table 1.

The melting enthalpy Δ*H*, temperatures characterizing the melting process, and the degree of crystallinity *X*_c_ of polymeric composites were determined on the basis of DSC thermograms. The analysis of the results focused mainly on DSC curves obtained from the first course of heating and cooling (Table 5). From the first measurement curve, it is possible to deduce the thermal history of the sample resulting from processing. Comparing in pairs the results for the extrudate obtained according to the experimental design layouts 10 and 12, 10 and 14, as well as 6 and 14 (Figure 14, Figure 15 and Figure 16), which were obtained by changing one of the process parameters, it can be stated that the processing affects the content of crystalline phase in the extrudate obtained, and thus the melting enthalpy and melting temperature range. However, the extrudate obtained according to the experimental design layouts 13 and 14 were obtained under the same processing conditions but with different filler content. Taking into account the values of *T*_m_ and *T*_c_ it can be concluded that talc is a filler that acts as a good nucleating agent, hence an increase in crystallinity of extrudates. The melting point of the obtained composites, expressed as the *T*_m_ parameter, is higher than that of the polypropylene from the manufacturer (Figure 17). The crystallisation temperatures *T*_c_ of extrudates are also higher than PP itself, indicating the presence of smaller polymer crystallites in the extrudate than in the initial PP. This is also confirmed by the melting peak width, which is smaller in the case of composite extrudates. The observed degrees of crystallinity of the extrudates are higher than for the initial PP, except for the extrudate obtained according to the experimental design layout 14. The observed degrees of crystallinity of the extrudates are higher than for the initial PP, except for the extrudate obtained according to the experimental design layout 14. A slightly lower *X*_c_ for the extrudate 14 is unexpected, but it can be the effect of the size of talc particles.

On the basis of the obtained results, it can also be concluded that the size of talc particles affects the degree of crystallinity of polypropylene—the greater the fineness, the higher the degree of crystallinity. This is confirmed by the highest crystallinity values in the samples obtained according to the experimental design layouts 12 and 10, for which the best filler fragmentation was found in the SEM photographs, which were the effect of trilobe kneading elements interlacing and closely overlapping in the intensive mixing zone. According to the predictions, the highest degree of crystallinity should be recorded for the extrudate sample 12, taking into account the significant screw rotational speed, which additionally increases the shear intensity of the plasticizing system and undoubtedly has a significant effect on the filler fragmentation. At such high rotational speed, however, the possibility of thermo-oxidative degradation must be taken into account, which in consequence leads to a decrease in the molecular weight of the polymer and deterioration of mechanical properties [14]. Negative changes in the structure of the matrix of the layout 12 itself confirm the measurement of the melt flow rate and the results obtained in the static tensile test, where the *MFR* value is higher and the tensile strength is lower than that of the layout 10 extruded at four times lower screw speed. Significantly lower values of crystallinity degree obtained for samples produced according to the experimental design layouts 6, 13, and 14 are the result of less effective talc fragmentation. Apart from the favourable phenomenon of nucleation of crystallites, filler particles can also limit the mobility of macromolecules and slow down the growth of crystallites due to their considerable size.

## 4. Conclusions

Corotating twin-screw extrusion is a complex process depending on a number of different material, technological and constructional factors. The phenomena occurring inside the plasticizing system have a significant influence on the properties of the obtained extrudate. It is difficult to unequivocally determine which of the analysed independent factors has the greatest influence on the examined properties, because the developed empirical models showed numerous statistically significant interactions between independent factors. Pareto charts of standardized effects show that the most statistically significant influence on mechanical properties and melt flow rate of the examined PP/talc composites is exerted by screw rotational speed and its interactions with other factors. It should be noted, however, that the influence of screw speed, or more precisely its significant values, on the properties is rather negative, as it causes the deterioration of tensile strength, decrease of crystallinity degree and increase of MFR value through the degradation of macromolecules caused by a significant gain in shear rate. The greatest difference in crystallinity degree was noted for the experimental design layouts, which differed in the distance between the kneading elements. This is due to the much lower efficiency of talc fragmentation by the layout with a greater distance between the elements and, consequently, a smaller number of potential nucleation sites of crystallites.

## Figures and Tables

**Table 1:** Experimental design and results of tests on the properties of the obtained extrudate—mean values with standard deviations.

Experimental Design Layout	α/^o^	*d/*mm	*n*/min^−1^	*u*/%	*MFR*/(g/10min)	σ/MPa	ε/%
1	0	0.5	100	10	1.24 ± 0.03	22.04 ± 0.63	6.50 ± 0.50
2	0	0.5	100	16	1.38 ± 0.04	20.14 ± 0.74	5.15 ± 0.20
3	0	0.5	400	10	1.50 ± 0.04	19.40 ± 0.31	5.38 ± 0.05
4	0	0.5	400	16	1.37 ± 0.02	19.26 ± 0.46	4.79 ± 0.20
5	0	4.5	100	10	1.28 ± 0.02	21.98 ± 0.35	6.86 ± 0.10
6	0	4.5	100	16	1.35 ± 0.02	21.40 ± 0.49	6.05 ± 0.19
7	0	4.5	400	10	1.37 ± 0.02	20.20 ± 0.66	5.80 ± 0.30
8	0	4.5	400	16	1.38 ± 0.02	19.92 ± 0.65	4.91 ± 0.11
9	60	0.5	100	10	1.31 ± 0.03	23.02 ± 0.91	6.36 ± 0.18
10	60	0.5	100	16	1.36 ± 0.03	22.36 ± 0.87	5.95 ± 0.41
11	60	0.5	400	10	1.37 ± 0.04	20.34 ± 0.32	5.72 ± 0.63
12	60	0.5	400	16	1.49 ± 0.02	18.58 ± 0.43	5.31 ± 0.23
13	60	4.5	100	10	1.32 ± 0.02	22.40 ± 1.06	6.21 ± 0.36
14	60	4.5	100	16	1.25 ± 0.02	23.54 ± 0.78	5.85 ± 0.68
15	60	4.5	400	10	1.44 ± 0.01	20.78 ± 0.65	6.25 ± 0.19
16	60	4.5	400	16	1.37 ± 0.03	20.20 ± 0.20	5.81 ± 0.14
17 (C)	30	2.5	250	13	1.36 ± 0.02	18.56 ± 0.48	6.54 ± 0.37

**Table 2:** Model of melt flow rate (MFR)–ANOVA table. *R ^2^*= 0.82; *R_adj_^2^* = 0.79.

Source of Variation	*SS*	*df*	*MS*	*F*	*p*
α	0.0003	1	0.0003	0.28	0.59730
*d*	0.0205	1	0.0205	18.03	0.00007
*n*	0.2000	1	0.2000	176.03	0.00000
*u*	0.0045	1	0.0045	3.96	0.05048
α*d*	0.0010	1	0.0010	0.86	0.35621
α*n*	0.0010	1	0.0010	0.86	0.35621
α*u*	0.0007	1	0.0007	0.63	0.42869
*dn*	0.0024	1	0.0024	2.13	0.14891
*du*	0.0180	1	0.0180	15.84	0.00017
*nu*	0.0205	1	0.0205	18.03	0.00007
α*dn*	0.0115	1	0.0115	10.14	0.00217
α*du*	0.0442	1	0.0442	38.89	0.00000
α*nu*	0.0480	1	0.0480	42.27	0.00000
*dnu*	0.0045	1	0.0045	3.96	0.05048
Error	0.0795	70	0.0011		
Total *SS*	0.4566	84			

*SS*—sum of squares; *df*—number of the degrees of freedom; *MS*—mean sum of squares; *F*—values of the test statistic; *p*—value of probability corresponding to the test statistic value.

**Table 3:** Model of tensile strength σ–ANOVA table. *R^2^* = 0.87; *R_adj_^2^* = 0.84 (markers as in Table 2).

Source of Variation	*SS*	*df*	*MS*	*F*	*p*
*curvature*	27.2745	1	27.2745	69.16	0.000000
α	13.9219	1	13.9219	35.30	0.000000
*d*	8.1291	1	8.1291	20.61	0.000025
*n*	97.2402	1	97.2402	246.56	0.000000
*u*	6.6640	1	6.6640	16.90	0.000113
α*d*	0.0009	1	0.0009	0.002	0.962247
α*n*	6.2968	1	6.2968	15.97	0.000167
α*u*	0.3443	1	0.3443	0.87	0.353622
*dn*	0.9214	1	0.9214	2.34	0.131237
*du*	5.0576	1	5.0576	12.82	0.000654
*nu*	0.1634	1	0.1634	0.41	0.522076
α*dn*	0.4522	1	0.4522	1.15	0.288209
α*du*	0.9804	1	0.9804	2.49	0.119721
α*nu*	7.0823	1	7.0823	17.96	0.000073
*dnu*	1.2725	1	1.2725	3.23	0.077107
Error	25.6355	65	0.3944		
Total *SS*	201.8958	80			

**Table 4:** Model of elongation at tensile strength ε—ANOVA table. *R ^2^*= 0.85; *R_adj_^2^*= 0.82 (markers as in Table 2).

Source of Variation	*SS*	*df*	*MS*	*F*	*p*
*curvature*	2.8020	1	2.8020	38.64	0.00000
*α*	0.6732	1	0.6732	9.28	0.00330
*d*	2.0598	1	2.0597	28.41	0.00000
*n*	7.4918	1	7.4918	103.33	0.00000
*u*	8.3347	1	8.3347	114.95	0.00000
α*d*	0.2997	1	0.2997	4.13	0.04600
α*n*	1.7326	1	1.7326	23.90	0.00001
α*u*	1.2498	1	1.2498	17.24	0.00010
*dn*	0.3757	1	0.3757	5.18	0.02602
*du*	0.0233	1	0.0233	0.32	0.57240
*nu*	0.4145	1	0.4145	5.72	0.01963
α*dn*	1.9635	1	1.9635	27.08	0.00000
α*du*	0.1813	1	0.1813	2.50	0.11852
α*nu*	0.0150	1	0.0150	0.21	0.65068
*dnu*	0.2668	1	0.2668	3.68	0.05933
Error	4.8579	67	0.0725		
Total *SS*	33.2901	82			

**Table 5:** Results of DSC measurements of melting temperature, melting enthalpy and degree of crystallinity of the initial PP and samples of the extrudate obtained according to the experimental design layouts (Table 1).

	I Heating					Cooling	II Heating		
Design Layout	*T*_m_/°C	Δ*H*/J/g	*X*_c_/%	*T*_m1_/°C	*T*_m2_/°C	*T*_c_/°C	*T*_m_/°C	Δ*H*/J/g	*X*_c_/%
PP	168	88	42.1	124	189	118	167	95	45.5
6	173	75	42.7	126	187	121	169	84	47.8
10	173	94	53.5	125	186	127	169	99	56.4
12	169	88	50.1	125	182	123	168	95	54.1
13	170	83	44.1	120	180	122	167	93	49.4
14	170	70	39.9	120	184	121	167	75	42.7

*T*_m1_—initial temperature of the melting process; *T*_m2_—end temperature of the melting process; *T*_m_—maximum temperature of the melting process; *T*_c_—crystallization temperature;Δ*H*—melting enthalpy; *X*_c_—crystallinity degree.
